# Nonessential Role for the NLRP1 Inflammasome Complex in a Murine Model of Traumatic Brain Injury

**DOI:** 10.1155/2016/6373506

**Published:** 2016-04-20

**Authors:** Thomas Brickler, Kisha Gresham, Armand Meza, Sheryl Coutermarsh-Ott, Tere M. Williams, Daniel E. Rothschild, Irving C. Allen, Michelle H. Theus

**Affiliations:** The Department of Biomedical Sciences and Pathobiology, Virginia-Maryland Regional College of Veterinary Medicine, Duck Pond Drive, Blacksburg, VA 24061, USA

## Abstract

Traumatic brain injury (TBI) elicits the immediate production of proinflammatory cytokines which participate in regulating the immune response. While the mechanisms of adaptive immunity in secondary injury are well characterized, the role of the innate response is unclear. Recently, the NLR inflammasome has been shown to become activated following TBI, causing processing and release of interleukin-1*β* (IL-1*β*). The inflammasome is a multiprotein complex consisting of nucleotide-binding domain and leucine-rich repeat containing proteins (NLR), caspase-1, and apoptosis-associated speck-like protein (ASC). ASC is upregulated after TBI and is critical in coupling the proteins during complex formation resulting in IL-1*β* cleavage. To directly test whether inflammasome activation contributes to acute TBI-induced damage, we assessed IL-1*β*, IL-18, and IL-6 expression, contusion volume, hippocampal cell death, and motor behavior recovery in* Nlrp1* ^−/−^,* Asc* ^−/−^, and wild type mice after moderate controlled cortical impact (CCI) injury. Although IL-1*β* expression is significantly attenuated in the cortex of* Nlrp1* ^−/−^ and* Asc* ^−/−^ mice following CCI injury, no difference in motor recovery, cell death, or contusion volume is observed compared to wild type. These findings indicate that inflammasome activation does not significantly contribute to acute neural injury in the murine model of moderate CCI injury.

## 1. Introduction

Mechanical trauma to the CNS results in the disruption of the cellular microenvironment leading to massive necrotic and apoptotic loss of neuronal and glia populations. The progressive cascade of secondary events, including ischemia, inflammation, excitotoxicity, and free radial release, contributes to neural tissue damage. Activation of the innate immune response, including microglia, peripheral-derived macrophages, and astrocytes [1–3], can lead to the expression of proinflammatory cytokines, chemokines, and reactive oxygen species, thereby triggering the inflammatory responses in the central nervous system (CNS). Recently, the initiation of such a response following tissue injury was shown to involve a multiprotein complex called the inflammasome [4]. This cytosolic complex enables the activation of proinflammatory caspases, mainly caspase-1 [5, 6], resulting in a potent inflammatory response. Inflammasome complexes generally have three main components: an NLR protein; the enzyme caspase-1; and an adaptor protein that facilitates the interaction between the two. The NOD-like receptors (NLRs) are critical in this process and members of the inflammasome forming NLR subfamily recruit the adapter apoptosis-associated speck-like protein (ASC) to activate caspase-1. Currently, at least 8 different NLR proteins are well characterized as being capable of inflammasome formation under a diverse range of stimuli. In the brain, the inflammasomes forming NLRs, NLRP1, NLRP2, and NLRP3 have each been shown to modulate caspase-1 activation and the subsequent processing of IL-1*β* and IL-18, primarily from glia cells [7–10]. The CNS is particularly sensitive to IL-1*β* and IL-18 signaling, since multiple neural cell types express receptors for these cytokines [11–13]. In addition, activated caspase-1 can mediate a form of necrotic cell death known as pyroptosis [14–16], making it a potential candidate for cell death signaling within neurons following injury. Therefore, induction of the NLR-mediated inflammasome complex could contribute to the proinflammatory milieu as well as neuronal pyroptosis following immunopathogenic conditions such as traumatic brain injury (TBI). Interestingly, recent studies have demonstrated assembly of the NLRP1- and NLRP3-inflammasome complex including increased expression of ASC, activation of caspase-1, and processing of IL-1*β* in a rat model of TBI [8, 9]. Furthermore, therapeutic administration of anti-ASC neutralizing antibodies was shown to reduce the innate immune response and significantly decrease contusion volume in the same model [9]. These studies suggest that inflammasome activation plays a critical role in acute neural injury and that pyropotosis may be a key element of neuronal cell death following brain trauma.

To better understand the role of the NLRP1 inflammasome complex in TBI-induced damage, we sought to evaluate the effects of NLRP1 and ASC gene deletion on cortical tissue loss in a murine model of controlled cortical impact (CCI) injury. This model produces a well-demarcated cortical lesion that mimics the contusions commonly observed in TBI patients. Because the pathophysiological sequela of TBI is dependent on impact severity and location, we investigated whether the absence of inflammasome activation impacts the histopathological outcome using this distinct model. The overall goal of this study was to assess the role of the NLR inflammasome following CCI injury by quantifying IL1-*β* and IL-18 expression and determine whether inflammasome disruption impacts cortical contusion volume and motor recovery in wild type,* Nlrp1* ^−/−^, and* Asc* ^−/−^ mice.

## 2. Materials and Methods

### 2.1. Animals/Ethics Statement

The* Nlrp1* ^−/−^ mice were provided by Dr. Beverly H. Koller (UNC Chapel Hill) and the* Asc* ^−/−^ (*Pycard* ^−/−^) mice were acquired from Genetech. The generation and characterization of* Nlrp1* ^−/−^ and* Asc* ^−/−^ mice have been previously described [17, 18]. All mice were maintained on the C57Bl/6 background and all animals were genotyped using standard PCR analysis prior to each study. All experiments were conducted in accordance with the NIH Guide for the Care and Use of Laboratory Animals and were conducted under the approval of the Virginia Tech Institutional Animal Care and Use Committee (IACUC; #12-081) and the Virginia-Maryland College of Veterinary Medicine.

### 2.2. CCI Injury

Male mice, 2–4 months old, were anesthetized with ketamine and xylazine by intraperitoneal (i.p.) injection and positioned in a stereotaxic frame [19, 20]. Body temperature was monitored with a rectal probe and maintained at 37°C with a controlled heating pad set. A 5 mm craniotomy was made using a portable drill over the right parietal-temporal cortex (−2.5 mm A/P and 2.0 mm lateral from bregma). Injury was induced by moderate CCI using the eCCI-6.3 device (Custom Design & Fabrication, Richmond, VA; 4 mm impounder) at a velocity of 3.5 m/s, depth of 0.5 mm, and 150 ms impact duration [19, 20]. Moderate CCI injury results in a well-demarcated cortical lesion or cavity that mimics the contusions commonly observed in TBI patients. Sham controls received anesthesia, skin incisions, and sutures only. Following injury, the incision was closed using Vetbond tissue adhesive (3M, St. Paul, MN, USA) and the animals were placed into a heated cage to maintain body temperature for 1 h after injury. At 1, 3, or 14 days after CCI injury, mice were anesthetized and brain tissue removal was performed following decapitation. Fresh frozen tissue was embedded in OCT and sectioned at 30 *μ*m thick. Five serial coronal sections were (300 *μ*m apart) stained for Nissl substance [21].

### 2.3. Rotarod Assessment

Motor function was evaluated using Rotarod testing, as previously described [21, 22]. Assessment was performed on the Economex (Columbus Instruments, Columbus, OH) at 3, 7, and 14 days after CCI by an observer unaware of experimental groups. The starting velocity was set at 10 rpm and accelerated to 0.1 rpm/sec. Animals were pretrained for 4 consecutive days prior to CCI injury with 3 trials (2 minutes resting in between) each day. Each trial ended when the animal fell off the Rotarod or gripped the rod and passively spun more than once. Time to fall was recorded for each trial per animal. A baseline (seconds) was collected on the fourth day of training. Evaluation of motor function after injury was based on individual scores relative to each animal's baseline latencies and represented as percentage of baseline.

### 2.4. Evaluation of Contusion Volume

Lesion or contusion volume was assessed by a blinded investigator using Cavalieri estimator from StereoInvestigator (MicroBrightField, Williston, VT, USA) and an Olympus BX51TRF motorized microscope (Olympus America, Center Valley, PA, USA). Contusion volume (mm^3^) was determined as previously described [21]. Briefly, volume analysis was performed by estimating the area of tissue loss in the ipsilateral cortical hemisphere for five coronal serial sections at or around the epicenter (−1.1 to −2.6 mm posterior from bregma) of injury. Nissl stained serial sections were viewed under brightfield illumination at a magnification of 4x. A random sampling scheme was used that estimates every 10th section from rostral to caudal, yielding five total sections to be analyzed. A randomly placed grid with 100 *μ*m spaced points was placed over the ipsilateral hemisphere and the area of contusion was marked within each grid. Contusion boundaries were identified by loss of Nissl staining, pyknotic neurons, and tissue hemorrhage. The contoured areas, using grid spacing, were then used to estimate total tissue volume based on section thickness, section interval, and total number of sections within the Cavalieri program, StereoInvestigator. Data is represented as volume of tissue loss or contusion volume (mm^3^) for wild type,* Nlrp1* ^−/−^, and* Asc* ^−/−^ mice.

### 2.5. Evaluation of Protein Cytokine Levels in Cortical Tissue Samples

Protein was isolated from cortical tissue samples of wild type,* Nlrp1* ^−/−^, and* Asc* ^−/−^ mice 1 day after CCI injury as previously described [21]. Briefly, fresh brain tissue was dissected in L15 (Gibco) media on ice and homogenized in RIPA buffer (pH 7.5, 1% NP-40, 1% sodium deoxycholate, 0.1% SDS, 0.15 M NaCl, 2 mM EDTA, and 0.01 M sodium phosphate) in the presence of complete protease inhibitor cocktail (Roche, Florence, SC, USA) and phosphatase inhibitor cocktail 2 (Sigma-Aldrich, St. Louis, MO, USA). Supernatant was collected by centrifuging at 14 000 ×g for 30 min at 4°C and the Lowry assay was used for the determination of protein concentration (Pierce, Rockford, IL, USA). Protein samples were then tested for IL-1*β*, IL-18, and IL-6 expression levels using ELISA (BD Biosciences), as previously described [23]. Final concentrations of each cytokine were calculated based on internal standard controls and represented as pg/mL, then normalized to the amount of protein (mg) loaded per well of the ELISA and represented at (pg/mL)/mg for each sample. This is a standard procedure to account for differences in starting protein levels that could significantly influence the final concentration of each cytokine [24–26].

### 2.6. Metadata Analysis

The microarray data was generated following a metadata analysis or data mining of publically available datasets using a publically accessible microarray meta-analysis NextBio search engine, available at http://www.nextbio.com/b/search/ba.nb. The data analysis was performed from the following datasets: human: GSE2392, 1432, 10612, 12837, 12305 12679; mouse: GSE17256, 10246, 9566, 11288; TBI study: GSE2392.

### 2.7. Statistical Analysis

Data was graphed using GraphPad Prism, version 4 (GraphPad Software, Inc., San Diego, CA). Student's two-tailed *t*-test was used for comparison of two experimental groups. Multiple comparisons were done using one-way and two-way ANOVA, where appropriate, followed by Tukey test for multiple pairwise examinations. Changes were identified as significant if *P* was less than 0.05. Mean values were reported together with the standard deviation (SD).

## 3. Results 

### 3.1. NLRP1 Inflammasome Activation Does Not Contribute to Acute Cortical Damage after CCI Injury

**Figure 1:**
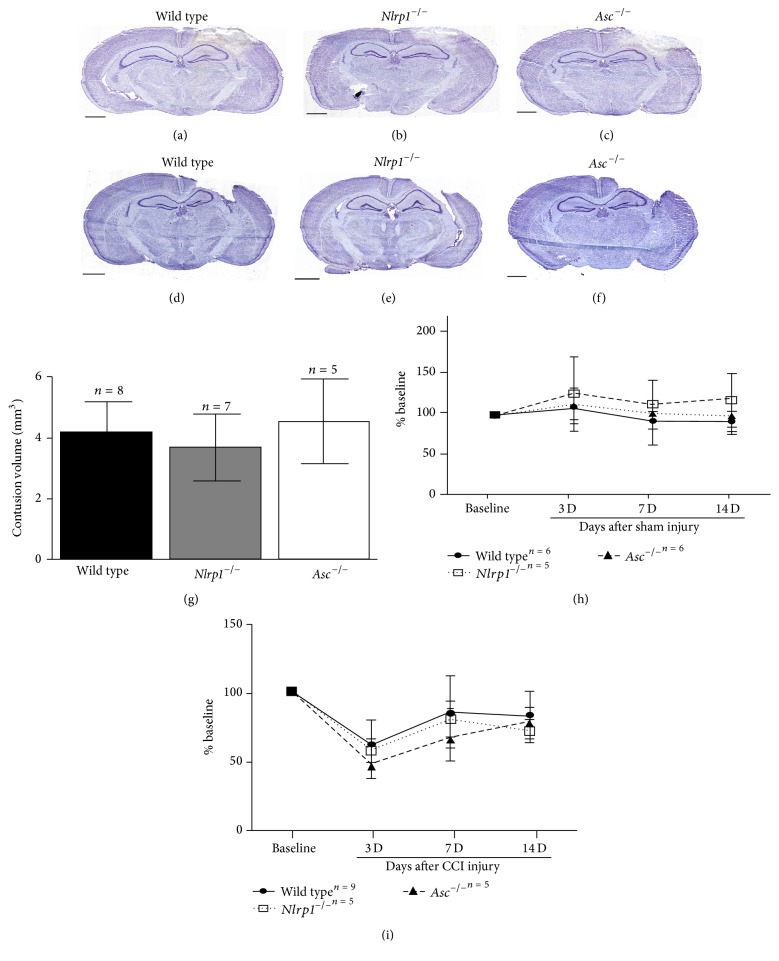
Genetic disruption of the NLRP1 inflammasome complex has no effect on contusion volume and motor deficits following CCI injury. Using Cavalieri estimator on Nissl stained sections collected from (a, d) wild type, (b, e)* Nlrp1* ^−/−^, and (c, f)* Asc* ^−/−^ brains at 3 and 14 days after injury, respectively, shows no significant change in contusion volume (mm^3^). (d) Bar graph represents mean contusion volume ± SD in wild type,* Nlrp1* ^−/−^, and* Asc* ^−/−^ mice (*n* = 5–8 per group). Rotarod assessment or motor function was performed in each group and demonstrates no significant difference between sham-injured mice (h) or CCI-injured mice (i). *n* = 5–9 per group, represented as mean ± SD.

Inflammasome complex formation has been shown to play a critical role in initiating inflammation in a variety of settings [27], though our understanding of its role in neuroinflammation is limited. Here, we sought to analyze the effects of inflammasome disruption on acute neural tissue damage and cytokine production following TBI. Specifically, we evaluated injury outcome in* Nlrp1* ^−/−^ and* Asc* ^−/−^ mice, using the controlled cortical impact (CCI) model [19–21], at 3 days after injury. Serial sections were subjected to Nissl staining and contusion boundaries were demarcated by loss of Nissl stain, pyknotic neurons, and tissue hemorrhage. Using the Cavalieri estimator, we found no significant difference in contusion volume (*F* = 1.37, *P* = 0.3) among wild type (4.22 ± 0.97 mm^3^; *n* = 8),* Nlrp1* ^−/−^ (3.70 ± 1.12 mm^3^ *n* = 7), and* Asc* ^−/−^ (4.57 ± 1.43 mm^3^; *n* = 5) mice at 3 days after CCI (Figures 1(a)–1(c) and 1(g)) or 14 days (*F* = 1.07; *P* = 0.49); (3.113 ± 0.85 mm^3^ *n* = 8; 3.0 ± 1.2 mm^3^ *n* = 5; 3.76 ± 0.66 mm^3^ *n* = 5, resp.) (Figures 1(d)–1(f)) after CCI injury. These results indicate that genetic ablation of specific genes known to be involved in the formation of the NLR inflammasome complex has no effect on neural tissue loss in the cortex following acute TBI. We also performed Rotarod behavioral analysis to test whether motor deficit and recovery were affected by inflammasome disruption following CCI injury. Mice were pretrained on the Rotarod 4 days prior to CCI injury then subjected to motor assessment at 3, 7, and 14 days after sham or CCI injury. Time to fall was recorded then normalized to the average baseline time for each mouse. No differences between groups were seen following sham injury for each time point tested (Figure 1(h)). Although motor deficits were observed following CCI injury, no difference between groups was observed in motor ability at 3 days (wild type 60.07% ± 18.4  *n* = 9;* Nlrp1* ^−*/*−^  55.7% ± 9.1  *n* = 5; *Asc* ^−*/*−^  45.44% ± 10.5  *n* = 5) compared to baseline, or at any other time point tested (Figure 1(i)). These data correlate with contusion volume estimates and confirm that inflammasome disruption has no effect on neural tissue loss or motor function after CCI injury.

**Figure 2:**
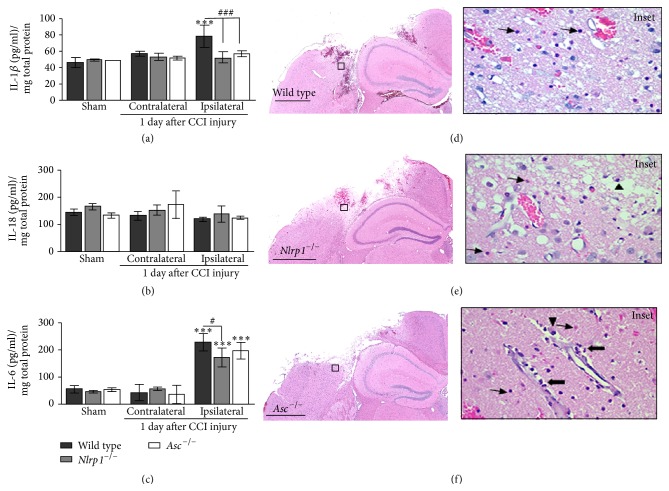
Cytokine protein expression and histopathology in wild type,* Nlrp1* ^−*/*−^, and* Asc* ^−*/*−^ mice. Quantification of IL-1*β*, IL-18, and IL-6 in wild type,* Nlrp1* ^−/−^, and* Asc* ^−/−^ cortical tissue samples analyzed by ELISA 1 day after CCI injury. (a) IL-1*β*, a direct release product of the inflammasome signaling cascade, is significantly reduced in the ipsilateral cortex of* Nlrp1* ^−/−^ and* Asc* ^−/−^ compared to wild type cortex. (b) No significant differences were observed in IL-18 levels 1 day after injury. (c) IL-6 is increased in wild type,* Nlrp1* ^−/−^, and* Asc* ^−/−^ mice following trauma with a slight attenuation in* Nlrp1* ^−/−^ ipsilateral cortex compared to wild type. ^*∗∗∗*^ *P* < 0.001 compared to the contralateral and sham-injured cortical control samples. ^#^ *P* < 0.05 and ^###^ *P* < 0.001 compared to wild type ipsilateral cortex. (d) H&E staining on coronal brain sections from wild type, (e)* Nlrp1* ^−*/*−^, and (f)* Asc* ^−*/*−^ mice shows no difference in histopathological outcome. Prominent cellular features present in all sections include large numbers of necrotic neurons (thin arrows), few macrophages (arrowheads), and neutrophils (thick arrows); insets. Scale bar = 1 mm. *n* = 4-5 per group, represented as mean ± SD.

Next, we investigated the protein expression of the proinflammatory cytokines IL-1*β*, IL-18, and IL-6 in the cortex of* Nlrp1* ^−/−^,* Asc* ^−/−^, and wild type mice following CCI injury. Following injury, we collected cortical tissue samples from the ipsilateral and contralateral hemispheres of sham- and CCI-injured mice at 24 hours. Total IL-1*β*, IL-18, and IL-6 levels were quantified using ELISA. Our findings demonstrate that CCI injury results in a significant increase in IL-1*β* (1.3-fold; 78.2 ± 13.6 pg/mL per mg protein) and IL-6 (5-fold; 229.3 ± 30.9) levels in the wild type CCI-injured ipsilateral cortex compared to uninjured contralateral (IL-1*β* 56.8 ± 2.9; IL-6 43.3 ± 29.2) or sham-injured ipsilateral tissue (IL-1*β* 45.3 ± 5.7; IL-6 56.6 ± 12.9) (Figures 2(a) and 2(c)). However, ipsilateral IL-1*β* levels are significantly attenuated in CCI-injured* Nlrp1* ^−/−^ (51.9 ± 6.5) and* Asc* ^−/−^ (56.8 ± 3.7) mice, where levels reach that of uninjured wild type samples (Figure 2(a)). On the other hand, we observed a similar increase in IL-6 in the injured cortex of wild type,* Nlrp1* ^−/−^, and* Asc* ^−/−^ mice (Figure 2(c)). Ipsilateral IL-6 levels are slightly reduced in* Nlrp1* ^−/−^ (172.6 ± 34.9) but not in* Asc* ^−/−^ (197.4 ± 29.7) compared to wild type. Interestingly, no significant difference in IL-18 levels was found following CCI injury, although a reduced trend in all ipsilateral samples is observed (Figure 2(b)). We find that disruption of the NLRP1 inflammasome complex leads to an attenuation of IL-1*β* production, an end-product of the inflammasome complex, while having minimal effects on IL-6 following CCI injury. These data indicate that IL-1*β* does not significantly contribute to the neural tissue injury in this model. We further characterized the histopathological changes induced by CCI injury in wild type (Figure 2(d)),* Nlrp1* ^−*/*−^ (Figure 2(e)), and* Asc* ^−*/*−^ (Figure 2(f)) using H&E staining. For each strain of mice, we found a well-demarcated area of oncosis, hemorrhage, and loss of neuropil at 3 days after CCI injury. In all sections tested, the surrounding devitalized brain tissue was vacuolated and contained numerous oncotic neurons (Figure 2 insets; thin arrows), low numbers of macrophages containing phagocytized erythrocytes and cellular debris (Figure 2 insets; arrowheads), and numerous neutrophils present in the perilesion site and perivascular spaces (Figure 2 insets; thick arrows). Histopathological assessments revealed no significant differences in inflammatory cell composition or quantity between the wild type,* Nlrp1* ^−*/*−^, and* Asc* ^−*/*−^ mice in our assessments 3 days after CCI injury. Likewise, no overt differences in the pathological phenotype were observed between the strains of mice following CCI injury.

**Figure 3:**
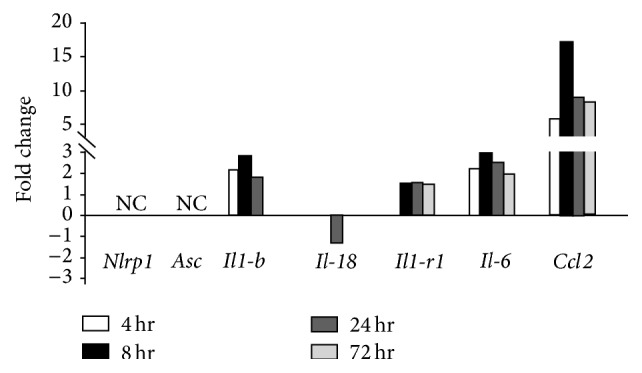
Time course of gene expression in the murine parietal cortex after CCI injury. Expression of several inflammasome related genes was compiled using the metadata-analysis system. Cortical tissue samples from sham- and CCI-injured mice were subjected to microarray analysis [28, 29]. Changes in expression are represented as fold change from uninjured levels. No change in* Nlrp1* or* Asc* was detected, while greater expression is noted for *Il-1b*,* Il-1r1*,* Il-6,* and* Ccl2 *at 4, 8, 24, or 72 hrs after trauma. Conversely, there was a reduced fold change (−1.28) in the expression of *Il-18* at 24 hours after CCI injury.

In order to gain more robust insight regarding gene expression patterns related to the NLRP1 inflammasome following CCI injury, we compared our ELISA data with previous microarray data obtained from murine cortical tissue samples following sham and CCI injury at 4–72 hrs after trauma using meta-analysis [28]. At each time point tested from the datasets GSE2392, no change was observed in gene expression for* Nlrp1* or* Asc* between sham and injury levels (Figure 3). However, in agreement with our ELISA results, the microarray metadata shows an increase in* Il1b* (1.8-fold) and* Il6* expression (2.54-fold) at 24 hr after CCI injury (Figure 3). Likewise,* Il1a* (data not shown; 8 hr after injury) and* Il1r1* (Figure 3) are also increased during acute trauma. Interestingly, at this time, there was also an observed decrease (−1.28-fold) in Il18 expression. We also observed that* Ccl2* expression consistently showed the highest fold increase at each time point tested (5.78-, 17.2-, 8.86-, and 5.59-fold, resp.). These results emphasize that trauma-induced changes occur at the transcriptional level in genes related to inflammasome associated pathways.

**Figure 4:**
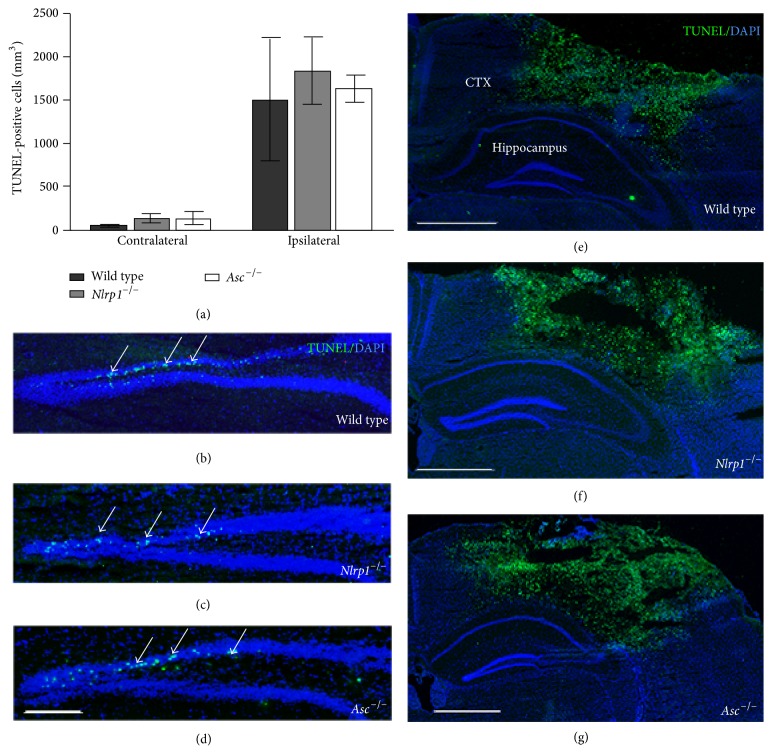
TUNEL analysis in the dentate gyrus and cortex of wild type,* Nlrp1* ^−*/*−^, and* Asc* ^−*/*−^ mice. (a) Quantification of TUNEL-positive cells in the ipsilateral and contralateral dentate gyrus (DG) 3 days following CCI injury. Increased cell death is observed in the ipsilateral compared to contralateral DG in all groups; however, no significant difference in TUNEL is observed between wild type,* Nlrp1* ^−/−^, and* Asc* ^−/−^ mice following trauma. Representative immunofluorescence images of TUNEL (green) DAPI (blue) colabeled cells in the ipsilateral DG and cortex of (b and e) wild type, (c and f)* Nlrp1* ^−/−^, and (d and g)* Asc* ^−/−^ mice, respectively. Images from 4x magnification; scale bar = 0.5 mm (b–d) and 1 mm (e–g). *n* = 4-5 per group, represented as mean ± SD.

Hippocampal dysfunction and cellular loss are the hallmark of TBI [21, 30–32]. In addition to cortical lesion volume, we evaluated cell death using terminal deoxynucleotidyl transferase dUTP nick end labeling (TUNEL) in the dentate gyrus (DG) and cortex of wild type,* Nlrp1* ^−*/*−^, and* Asc* ^−*/*−^ mice (Figure 4). Serial coronal sections were stained with TUNEL and optical fractionator, and StereoInvestigator was used to quantify the total number of TUNEL-positive cells in the contralateral and ipsilateral DG at 3 days after CCI. In all three strains, our analysis revealed a similar increase in cell death in the ipsilateral DG compared to contralateral; however, no significant difference was observed (Figure 4(a)) between wild type (1507.7 ± 1221.2),* Nlrp1* ^−*/*−^ (1840.6 ± 862.3), and* Asc* ^−*/*−^ (1635.8 ± 315.8) mice in the ipsilateral DG (Figures 4(b), 4(c), and 4(d), resp.). Similarly, no differences in TUNEL were seen in the cortex between the experimental groups (Figures 4(e)–4(g)). These findings indicate that the extent of cell death induced by cortical impact injury was not affected by the loss of Nlrp1 or Asc expression.

### 3.2. Meta-Analysis of* NLRP1, ASC, *and* IL-1β* Gene Expression

**Figure 5:**
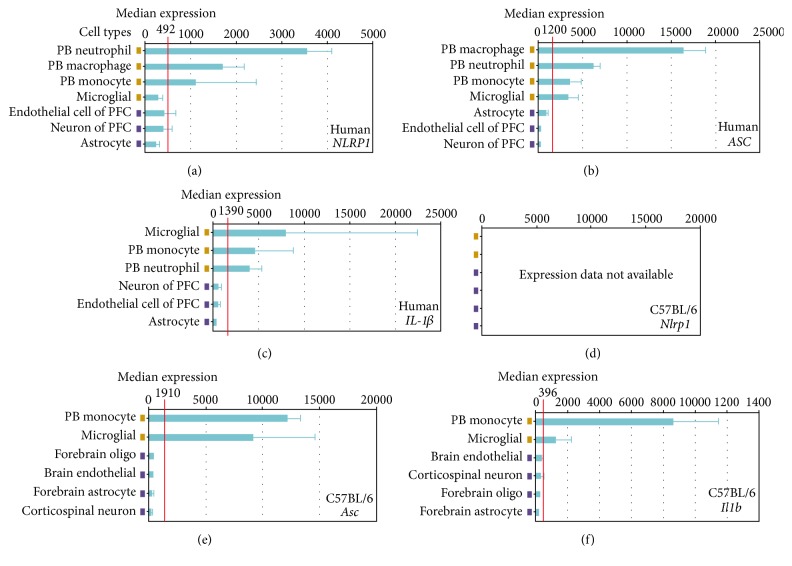
Cell-specific gene expression of* NLRP1*,* ASC*, and* IL-1β* in human and murine tissue. Expression of human (a)* NLRP1*, (b)* ASC,* and (c)* IL-1β* in relevant immune and neural system cell types was compiled using a publically accessible microarray meta-analysis search engine (NextBio website: http://www.nextbio.com/b/search/ba.nb. Accessed on September 8, 2014). Expression of mouse (d)* Nlrp1*, (e)* Asc,* and (f)* Il1b *from the C57Bl/6 strain can be directly compared to the human expression profile in specific immune and neural cells. No gene expression data was available for* Nlrp1* (*Nlrp1a, Nlrp1b, and Nlrp1c*) at the time the search engine was accessed. PB: peripheral blood; PFC: prefrontal cortex.

Prior studies have shown that therapeutic targeting of the NLRP1 inflammasome attenuates the immune response and significantly improves histopathologic features associated with traumatic brain injury in rats (PMID: 19401709; 22781337). Likewise, the inflammasome has been suggested to be a promising target for therapeutic development in humans to treat a variety of conditions, including following CNS injury (reviewed in 26024799). Due to the therapeutic interest in targeting the NLR inflammasome, we sought to evaluate cell-specific expression in murine and human samples using a data mining bioinformatics approach.* NLRP1*,* ASC,* and* IL-1β* expression were analyzed in immune and neural cell types from both humans and mice using a publically accessible microarray meta-analysis search engine (NextBio website, available at http://www.nextbio.com/b/search/ba.nb), as previously described [29]. This analysis revealed highly variable levels between select cell populations relevant to the immune response compared to neural tissue. Overall, human* NLRP1, ASC, *and* IL-1β* show greater median cell expression in the naïve peripheral blood (PB) cells known to respond to trauma. These include neutrophils, macrophages, and monocytes (Figures 5(a)–5(c)), with the exception of* IL-1β*, which has the highest level of expression in human microglial cells (Figure 5(c)). Brain-derived cell types show lower median expression levels of these genes suggesting that the greatest activity occurs in response to immune activation.* Nlrp1 *(*Nlrp1a; Nlrp1b; Nlrp1c*) (Figure 5(d)) expression data has not been added to the datasets evaluated using this method. However, similar results to the human findings were obtained for* Asc* and* Il1-b* in naïve C57Bl/6 mice (Figures 5(e) and 5(f), resp.).

## 4. Discussion 

New insights into the role of the NLR inflammasome complex during acute inflammation have prompted its investigation in the pathogenesis of numerous neurological diseases, including TBI [33, 34]. The current study shows that genetic deletion of NLRP1 (*Nlrp1a, Nlrp1b*, and* Nlrp1c*) or ASC, essential proteins for the assembly of the NLRP1 inflammasome complex, has no direct effect on cortical tissue loss, hippocampal cell death, or motor behavior deficits. In the present study, we utilized the Rotarod as a measure of functional recovery following CCI injury in the motor cortex. This technique measures aspects of motor impairment that are not evident by either the beam-balance or beam-walking tasks in our model. For this reason our current experiments focused on the Rotarod, which is the most robust, sensitive, and efficient index for assessing motor impairment produced by CCI injury [22]. Although we find a significant attenuation of IL-1*β* in* Nlrp1* ^−/−^ or* Asc* ^−/−^ mice, histopathological changes seen following cortical trauma were similar to that found in wild type mice. These results are the first to identify a nonessential role for the NLR inflammasome in injury outcome following controlled cortical impact using a genetic approach. Our data is not consistent with previous findings that demonstrate significant protection following administration of ASC neutralizing antibodies in the rat lateral fluid percussion injury (FPI) model of TBI [9]. This discrepancy may be due to species differences between rats and mice or possible variations in the cytokine profile induced between the CCI and fluid percussion TBI models. Likewise, recent studies indicate that mouse and rat genetic factors may also mediate some of this variability [35]. It is also possible that other compensatory mechanisms may be associated with TBI progression in the* Nlrp1* ^−*/*−^ and* Asc* ^−*/*−^ mice, which are completely devoid of NLRP1 inflammasome function from birth. The discrepancies observed in our model versus the prior studies in rats underscore the need for further investigation to develop additional mechanistic insight into the role of the NLRP1 inflammasome following traumatic brain injury.

In the present study, we utilized NLRP1 and ASC gene targeted knockout mice, which have been previously shown to prevent NLRP1-mediated inflammasome complex formation during acute inflammation [17, 18, 36]. We demonstrate attenuated inflammasome function in the* Nlrp1* ^−/−^ and* Asc* ^−/−^ mice, as evidenced by reduction of IL-1*β* following acute CCI injury. Interestingly, IL-18 levels were unaffected in the cortex after trauma (Figure 2(b)). In fact, we observed a trend towards reduced IL-18 levels in wild type,* Nlrp1* ^−/−^, and* Asc* ^−/−^ mice indicating that noninflammasome regulated pathways may be acting to suppress IL-18 induction in response to cortical impact. This is further supported by our metadata analysis that also shows reduced expression of* Il-18* at the transcription level (Figure 3). Overall, we find that abolishing IL-1*β* expression in the absence of ASC and NLRP1 does not correlate with changes in lesion volume or behavioral outcome after controlled cortical impact. Therefore, in order to identify key cytokines that may play a more central role, we further analyzed IL-6, as this cytokine has been largely implicated in tissue damage and progression of cavity formation. IL-6 generation has also been shown to be significantly modulated by several NLRs and is often dysregulated in NLR deficient mice in other models beyond the nervous system [36–38]. Compared to the ~2-fold increase in IL-1*β*, there was a ~6-fold increase in IL-6 expression after TBI implying that this protein, among others, is more central to the inflammatory response in brain trauma. NLRP1 has not previously been shown to regulate IL-6 and the small reduction seen in the cortical tissue samples of TBI-injured* Nlrp1* ^−/−^ mice (Figure 2(c)) is somewhat surprising. We do not believe such minimal changes would impact the overall downstream effects on IL-6 production. However, it is possible that earlier induction (6–12 hrs) of IL-6 is unaffected, which is also critical for stimulating the inflammatory milieu and progression of injury.

ASC, NLRP1, and IL-1*β* are expressed in neurons and glial, vascular endothelial and peripheral-derived immune cells [39, 40] and have been shown to be upregulated after TBI [8, 9]. These immune mediators play a vital role in activating the inflammatory response, which is a necessary component of repair and healing [41, 42]. However, acute inflammation also exacerbates tissue damage in the brain for which IL-1 has been implicated as a major player [43, 44]. Previous studies in ischemic stroke have demonstrated that exogenous IL-1 administration can exacerbate neuronal injury [45], while inhibition of caspase-1 or IL-1 receptor provides protection [46, 47]. Deletion of IL-1*α* and IL-1*β* also can significantly reduce ischemic injury in mice [48]. Furthermore, current clinical trials of IL-1 receptor antagonist are underway in patients who suffer acute stroke [49]. However, our studies indicate that attenuation of IL-1*β* does not correlate with neuroprotection in CCI-injured* Nlrp1* ^−/−^ and* Asc* ^−/−^ mice suggesting that NLR inflammasome activation and IL-1 production may not play a significant role in neuronal damage after TBI. Injury-induced expression of IL-1*β* in the current model may be too weak to participate in eliciting a majority of the immune response. Our data shows that IL-1*β* is minimally upregulated at 24 hr (1.3-fold increase) as compared to IL-6 (5-fold increase) suggesting that other proinflammatory pathways may play a more prominent role [50]. For example, TNF expression is consistently upregulated across several TBI models in rodents including CCI, FPI, and stab wound injury, with detectable levels at 1 h after injury, maximal concentration at 3–8 h, and a decline at 24 h after injury [51, 52]. Similarly, IL-6 is also an important mediator of neuroinflammation in the brain [53, 54] suggesting that these and other cytokine pathways may predominate following CCI injury. Indeed, human clinical studies have demonstrated that levels of TNF, IL-6, IL-8, and IL-10 correlate with TBI severity and rates of complication [55–58].

We conclude that disruption of the NLRP1 inflammasome has no effect on injury outcome in the murine moderate CCI model. Inflammasome activation and subsequent IL-1*β* expression are not a limiting factor in the behavioral deficits, neuronal loss in the cortex, or hippocampus associated with acute CCI injury. NLR inflammasomes have been shown to be involved in a diverse range of conditions associated with aberrant inflammation, including many neurological and neurodegenerative conditions. While our current negative findings using a genetic approach were unexpected, they emphasize the need to further explore the clinical relevance and mechanistic details underlying the NLRP1 inflammasome in brain injury and other related central nervous system disorders.

## References

[1] Rivest S. (2009). Regulation of innate immune responses in the brain. *Nature Reviews Immunology*.

[2] Loane D. J., Byrnes K. R. (2010). Role of microglia in neurotrauma. *Neurotherapeutics*.

[3] Chen S., Pickard J. D., Harris N. G. (2003). Time course of cellular pathology after controlled cortical impact injury. *Experimental Neurology*.

[4] Martinon F., Burns K., Tschopp J. (2002). The Inflammasome: a molecular platform triggering activation of inflammatory caspases and processing of proIL-*β*. *Molecular Cell*.

[5] Chen S., Sun B. (2013). Negative regulation of NLRP3 inflammasome signaling. *Protein & Cell*.

[6] Rathinam V. A. K., Vanaja S. K., Fitzgerald K. A. (2012). Regulation of inflammasome signaling. *Nature Immunology*.

[7] Minkiewicz J., de Rivero Vaccari J. P., Keane R. W. (2013). Human astrocytes express a novel NLRP2 inflammasome. *Glia*.

[8] Liu H.-D., Li W., Chen Z.-R. (2013). Expression of the NLRP3 inflammasome in cerebral cortex after traumatic brain injury in a rat model. *Neurochemical Research*.

[9] de Rivero Vaccari J. P., Lotocki G., Alonso O. F., Bramlett H. M., Dietrich W. D., Keane R. W. (2009). Therapeutic neutralization of the NLRP1 inflammasome reduces the innate immune response and improves histopathology after traumatic brain injury. *Journal of Cerebral Blood Flow and Metabolism*.

[10] Jha S., Srivastava S. Y., Brickey W. J. (2010). The inflammasome sensor, NLRP3, regulates CNS inflammation and demyelination via caspase-1 and interleukin-18. *The Journal of Neuroscience*.

[11] Alboni S., Montanari C., Benatti C. (2014). Interleukin 18 activates MAPKs and STAT3 but not NF-*κ*B in hippocampal HT-22 cells. *Brain, Behavior, and Immunity*.

[12] Crampton S. J., Collins L. M., Toulouse A., Nolan Y. M., O'Keeffe G. W. (2012). Exposure of foetal neural progenitor cells to IL-1*β* impairs their proliferation and alters their differentiation—a role for maternal inflammation?. *Journal of Neurochemistry*.

[13] Miyoshi K., Obata K., Kondo T., Okamura H., Noguchi K. (2008). Interleukin-18-mediated microglia/astrocyte interaction in the spinal cord enhances neuropathic pain processing after nerve injury. *The Journal of Neuroscience*.

[14] Adamczak S. E., de Rivero Vaccari J. P., Dale G. (2014). Pyroptotic neuronal cell death mediated by the AIM2 inflammasome. *Journal of Cerebral Blood Flow and Metabolism*.

[15] Bergsbaken T., Fink S. L., Cookson B. T. (2009). Pyroptosis: host cell death and inflammation. *Nature Reviews Microbiology*.

[16] Zhang W.-H., Wang X., Narayanan M. (2003). Fundamental role of the Rip2/caspase-1 pathway in hypoxia and ischemia-induced neuronal cell death. *Proceedings of the National Academy of Sciences of the United States of America*.

[17] Kovarova M., Hesker P. R., Jania L. (2012). NLRP1-dependent pyroptosis leads to acute lung injury and morbidity in mice. *The Journal of Immunology*.

[18] Mariathasan S., Hewton K., Monack D. M. (2004). Differential activation of the inflammasome by caspase-1 adaptors ASC and Ipaf. *Nature*.

[19] Baumann G., Travieso L., Liebl D. J., Theus M. H. (2013). Pronounced hypoxia in the subventricular zone following traumatic brain injury and the neural stem/progenitor cell response. *Experimental Biology and Medicine*.

[20] Theus M. H., Ricard J., Bethea J. R., Liebl D. J. (2010). EphB3 limits the expansion of neural progenitor cells in the subventricular zone by regulating p53 during homeostasis and following traumatic brain injury. *Stem Cells*.

[21] Theus M. H., Ricard J., Glass S. J., Travieso L. G., Liebl D. J. (2014). EphrinB3 blocks EphB3 dependence receptor functions to prevent cell death following traumatic brain injury. *Cell Death & Disease*.

[22] Hamm R. J., Pike B. R., O'Dell D. M., Lyeth B. G., Jenkins L. W. (1994). The rotarod test: an evaluation of its effectiveness in assessing motor deficits following traumatic brain injury. *Journal of Neurotrauma*.

[23] Gris D., Ye Z., Iocca H. A. (2010). NLRP3 plays a critical role in the development of experimental autoimmune encephalomyelitis by mediating Th1 and Th17 responses. *The Journal of Immunology*.

[24] Allen I. C., Tekippe E. M., Woodford R.-M. T. (2010). The NLRP3 inflammasome functions as a negative regulator of tumorigenesis during colitis-associated cancer. *The Journal of Experimental Medicine*.

[25] Allen I. C., Wilson J. E., Schneider M. (2012). NLRP12 suppresses colon inflammation and tumorigenesis through the negative regulation of noncanonical NF-*κ*B signaling. *Immunity*.

[26] Williams T. M., Leeth R. A., Rothschild D. E. (2015). Caspase-11 attenuates gastrointestinal inflammation and experimental colitis pathogenesis. *American Journal of Physiology—Gastrointestinal and Liver Physiology*.

[27] Pedra J. H., Cassel S. L., Sutterwala F. S. (2009). Sensing pathogens and danger signals by the inflammasome. *Current Opinion in Immunology*.

[28] Natale J. E., Ahmed F., Cernak I., Stoica B., Faden A. I. (2003). Gene expression profile changes are commonly modulated across models and species after traumatic brain injury. *Journal of Neurotrauma*.

[29] Kupershmidt I., Su Q. J., Grewal A. (2010). Ontology-based meta-analysis of global collections of high-throughput public data. *PLoS ONE*.

[30] Levin H. S. (1998). Cognitive function outcomes after traumatic brain injury. *Current Opinion in Neurology*.

[31] Hamm R. J., Dixon C. E., Gbadebo D. M. (1992). Cognitive deficits following traumatic brain injury produced by controlled cortical impact. *Journal of Neurotrauma*.

[32] Hall E. D., Sullivan P. G., Gibson T. R., Pavel K. M., Thompson B. M., Scheff S. W. (2005). Spatial and temporal characteristics of neurodegeneration after controlled cortical impact in mice: more than a focal brain injury. *Journal of Neurotrauma*.

[33] Walsh J. G., Muruve D. A., Power C. (2014). Inflammasomes in the CNS. *Nature Reviews Neuroscience*.

[34] Fan L., Young P. R., Barone F. C., Feuerstein G. Z., Smith D. H., McIntosh T. K. (1995). Experimental brain injury induces expression of interleukin-1 beta mRNA in the rat brain. *Brain Research Molecular Brain Research*.

[35] Mills C. D., Hains B. C., Johnson K. M., Hulsebosch C. E. (2001). Strain and model differences in behavioral outcomes after spinal cord injury in rat. *Journal of Neurotrauma*.

[36] Williams T. M., Leeth R. A., Rothschild D. E. (2015). The NLRP1 inflammasome attenuates colitis and colitis-associated tumorigenesis. *Journal of Immunology*.

[37] Allen I. C., Moore C. B., Schneider M. (2011). NLRX1 protein attenuates inflammatory responses to infection by interfering with the RIG-I-MAVS and TRAF6-NF-*κ*B signaling pathways. *Immunity*.

[38] Allen I. C., Scull M. A., Moore C. B. (2009). The NLRP3 inflammasome mediates in vivo innate immunity to influenza A virus through recognition of viral RNA. *Immunity*.

[39] Lechan R. M., Toni R., Clark B. D. (1990). Immunoreactive interleukin-1*β* localization in the rat forebrain. *Brain Research*.

[40] Boutin H., Kimber I., Rothwell N. J., Pinteaux E. (2003). The expanding interleukin-1 family and its receptors: do alternative IL-1 receptor/signaling pathways exist in the brain. *Molecular Neurobiology*.

[41] Shechter R., London A., Varol C. (2009). Infiltrating blood-derived macrophages are vital cells playing an anti-inflammatory role in recovery from spinal cord injury in mice. *PLoS Medicine*.

[42] Popovich P. G., Longbrake E. E. (2008). Can the immune system be harnessed to repair the CNS?. *Nature Reviews Neuroscience*.

[43] Allan S. M., Tyrrell P. J., Rothwell N. J. (2005). Interleukin-1 and neuronal injury. *Nature Reviews Immunology*.

[44] Brough D., Tyrrell P. J., Allan S. M. (2011). Regulation of interleukin-1 in acute brain injury. *Trends in Pharmacological Sciences*.

[45] Yamasaki Y., Matsuura N., Shozuhara H. (1995). Interleukin-1 as a pathogenetic mediator of ischemic brain damage in rats. *Stroke*.

[46] Relton J. K., Rothwell N. J. (1992). Interleukin-1 receptor antagonist inhibits ischaemic and excitotoxic neuronal damage in the rat. *Brain Research Bulletin*.

[47] Hara H., Friedlander R. M., Gagliardini V. (1997). Inhibition of interleukin 1*β* converting enzyme family proteases reduces ischemic and excitotoxic neuronal damage. *Proceedings of the National Academy of Sciences of the United States of America*.

[48] Boutin H., LeFeuvre R. A., Horai R., Asano M., Iwakura Y., Rothwell N. J. (2001). Role of IL-1alpha and IL-1beta in ischemic brain damage. *The Journal of Neuroscience*.

[49] Emsley H. C. A., Smith C. J., Georgiou R. F. (2005). A randomised phase II study of interleukin-1 receptor antagonist in acute stroke patients. *Journal of Neurology, Neurosurgery and Psychiatry*.

[50] Namas R., Ghuma A., Hermus L. (2009). The acute inflammatory response in trauma/hemorrhage and traumatic brain injury: current state and emerging prospects. *Libyan Journal of Medicine*.

[51] Fan L., Young P. R., Barone F. C., Feuerstein G. Z., Smith D. H., McIntosh T. K. (1996). Experimental brain injury induces differential expression of tumor necrosis factor-*α* mRNA in the CNS. *Molecular Brain Research*.

[52] Kita T., Liu L., Tanaka N., Kinoshita Y. (1997). The expression of tumor necrosis factor-*α* in the rat brain after fluid percussive injury. *International Journal of Legal Medicine*.

[53] Shohami E., Novikov M., Bass R., Yamin A., Gallily R. (1994). Closed head injury triggers early production of TNF*α* and IL-6 by brain tissue. *Journal of Cerebral Blood Flow and Metabolism*.

[54] Taupin V., Toulmond S., Serrano A., Benavides J., Zavala F. (1993). Increase in IL-6, IL-1 and TNF levels in rat brain following traumatic lesion. Influence of pre- and post-traumatic treatment with Ro5 4864, a peripheral-type (p site) benzodiazepine ligand. *Journal of Neuroimmunology*.

[55] Cinat M. E., Waxman K., Granger G. A., Pearce W., Annas C., Daughters K. (1994). Trauma causes sustained elevation of soluble tumor necrosis factor receptors. *Journal of the American College of Surgeons*.

[56] Hensler T., Sauerland S., Bouillon B. (2002). Association between injury pattern of patients with multiple injuries and circulating levels of soluble tumor necrosis factor receptors, interleukin-6 and interleukin-10, and polymorphonuclear neutrophil elastase. *Journal of Trauma-Injury Infection & Critical Care*.

[57] Martin C., Boisson C., Haccoun M., Thomachot L., Mege J.-L. (1997). Patterns of cytokine evolution (tumor necrosis factor-*α* and interleukin-6) after septic shock, hemorrhagic shock, and severe trauma. *Critical Care Medicine*.

[58] Narayan R. K., Michel M. E., Ansell B. (2002). Clinical trials in head injury. *Journal of Neurotrauma*.

